# Expanding horizons in the management of IgA nephropathy with targeted therapies

**DOI:** 10.1016/j.kisu.2025.12.001

**Published:** 2026-07-20

**Authors:** Brad H. Rovin

**Affiliations:** 1 Division of Nephrology, The Ohio State University Wexner Medical Center, Columbus, Ohio, USA

**Keywords:** end-stage kidney disease, 4-hit hypothesis, IgA nephropathy, kidney failure, targeted therapy

It is fair to say that the management of IgA nephropathy (IgAN) is in the midst of a revolution. Although I could have used the word *transformation*, I think *revolution* is appropriate and will elaborate shortly.

Several conditions came together a few years ago to create a perfect incubator for the IgAN revolution. A key element was the publication of a white paper by a group interested in finding a pathway forward in drug development for this rare disease.^1^ This group was assembled under the auspices of the Kidney Health Initiative and was made up of experts in glomerular diseases, statistics, epidemiology, clinical trial design, drug development, and, critically, regulatory officials from the US Food and Drug Administration.^1^ The group reviewed all available data and concluded that proteinuria reduction could be a surrogate end point for accelerated approval of novel IgAN therapeutics if followed by continued monitoring to demonstrate a beneficial effect on glomerular filtration rate over 2 years. Using this framework, the nephrology and pharmaceutical communities have worked together to design clinical trials that examine proteinuria reduction. The reduction was generally measured after 9 months of therapy with a novel drug added to optimized inhibition of the renin-angiotensin system in patients with controlled blood pressure and compared with placebo or active control. Following this, patients were observed for a further 15 months of follow-up while maintaining the original blinded conditions, to assess effects on glomerular filtration rate.^2^, ^3^, ^4^, ^5^ This formula has proven to be a winner, with 2 drugs fully approved by the US Food and Drug Administration for IgAN,^6^^,^^7^ a further 3 drugs having achieved accelerated approval, and several drugs under active investigation.^8^, ^9^, ^10^

The second key element that has helped the IgAN revolution incubate is a deeper understanding of disease pathophysiology. Research into IgAN culminated in a disease pathway commonly called the “4-hit hypothesis.”^11^ The 4-hit hypothesis acknowledges the role of the mucosal immune system in overproduction of galactose-deficient IgA1 (the “antigen”).^11^ It also describes the role of antibodies against galactose-deficient IgA1 (or its self-aggregating properties) to form immune complexes or immune aggregates.^11^ These aggregates, possibly coupled to the soluble IgA Fc receptor cluster of differentiation (CD) 89, subsequently accumulate in the glomerular mesangium and initiate kidney injury after being taken up by the mesangial IgA receptor CD71.^11^ This injury occurs through a variety of proinflammatory mechanisms, including complement activation; immune cell recruitment; and expression of vasoactive factors, growth factors, and cytokines by glomerular cells and infiltrating leukocytes.^12^^,^^13^ The 4-hit hypothesis provides a convenient way to match drugs to pathway components with the expectation that, if these components are blocked, the disease could be controlled.

The final element that stoked the IgAN revolution is the increasing awareness that IgAN is not a slow-moving and benign glomerular disease.^14^ Epidemiologic studies have started to demonstrate that given a long enough follow-up, many patients with IgAN will go on to end-stage kidney disease, and many more will live with advanced chronic kidney disease and all its associated comorbidities.^14^^,^^15^ Even more disturbing has been the discovery that what nephrologists have held to be a ground truth for decades in the management of IgAN (i.e., that reduction of proteinuria to ≤1 g/d was good enough) does not appear to be good enough.^14^^,^^15^

So why a revolution? The nephrology community has been slow to embrace the new therapies and therapeutic goals, with many adopting a “wait-and-see” approach. The community as a whole appears content to manage with usual care (renin-angiotensin system inhibition) for months before considering a more specific treatment, possibly at the expense of ongoing nephron injury and loss. Going from no treatments besides renin-angiotensin system inhibitors and systemic corticosteroids to a paradigm that suggests patients with IgAN may require multiple therapies in parallel to control the immunologic disease, kidney inflammation, and chronic kidney disease is proving to be a difficult adjustment. A further challenge for the community is the realization that treatment targets may be more stringent and more encompassing than simply reducing proteinuria. The IgAN revolution is not just about developing new drugs, but a push to disrupt conventional thinking and bring forward new ideas.

To this end, experts in the care of patients with IgAN have been brought together to help facilitate the IgAN revolution through evidence-based education. In the following supplement, the reader will find an up-to-date discussion of the pathophysiology of IgAN, and an overview of current management practices and the importance of immunomodulation in avoiding progression to kidney failure, with appropriate cautions for close patient follow-up to monitor for the added risk of infection from immunomodulation. They will also find a review of newly approved therapies and drugs in the IgAN pipeline, with discussions of how therapies may be applied now. The supplement also explores what additional evidence will be needed to better understand how to use these new medications appropriately and how best to individualize patient management, including the identification and application of biomarkers of disease activity and response.

## Disclosure

This article is published as part of a supplement sponsored by Calliditas Therapeutics, an Asahi Kasei company.

BHR reports institutional grants from Biogen, the National Institutes of Health, and the Lupus Research Alliance; he has received consulting fees from Alexion Pharmaceuticals, Alpine Pharma, BioCryst Pharmaceuticals, Calliditas Therapeutics, Novartis, Q32 Bio, Omeros, Otsuka Pharmaceuticals, Travere Therapeutics, and Vera Therapeutics. He also has a leadership role at NephroNet, the Lupus Accelerating Breakthroughs Consortium/Lupus Research Alliance, and the Lupus Foundation of America.
